# Signature-scoring methods developed for bulk samples are not adequate for cancer single-cell RNA sequencing data

**DOI:** 10.7554/eLife.71994

**Published:** 2022-02-25

**Authors:** Nighat Noureen, Zhenqing Ye, Yidong Chen, Xiaojing Wang, Siyuan Zheng

**Affiliations:** 1 Greehey Children’s Cancer Research Institute, UT Health San Antonio San Antonio United States; 2 Department of Population Health Sciences, UT Health San Antonio San Antonio United States; International Laboratory for Human Genome Research Mexico; Weizmann Institute of Science Israel

**Keywords:** single cell RNA sequencing, signature scoring, benchmarking, gene counts, cancer stemness, Human

## Abstract

Quantifying the activity of gene expression signatures is common in analyses of single-cell RNA sequencing data. Methods originally developed for bulk samples are often used for this purpose without accounting for contextual differences between bulk and single-cell data. More broadly, few attempts have been made to benchmark these methods. Here, we benchmark five such methods, including single sample gene set enrichment analysis (ssGSEA), Gene Set Variation Analysis (GSVA), AUCell, Single Cell Signature Explorer (SCSE), and a new method we developed, Jointly Assessing Signature Mean and Inferring Enrichment (JASMINE). Using cancer as an example, we show cancer cells consistently express more genes than normal cells. This imbalance leads to bias in performance by bulk-sample-based ssGSEA in gold standard tests and down sampling experiments. In contrast, single-cell-based methods are less susceptible. Our results suggest caution should be exercised when using bulk-sample-based methods in single-cell data analyses, and cellular contexts should be taken into consideration when designing benchmarking strategies.

## Introduction

Single-cell RNA sequencing (scRNA-seq) is a powerful technology to study the ecosystems of normal and disease tissues. Gene expression signatures can be used to interrogate single cells for cell identities and other cellular properties (Noureen et al., 2021a) using signature-scoring methods. A key consideration for these methods is how to account for the variability of expression within the signature, a characteristic often associated with the overall expression variability of the interrogated sample. Despite widespread use and their benchmarking on certain applications (Holland et al., 2020; Zhang et al., 2020), limitations of these methods, including some initially developed for bulk samples, are incompletely understood in scRNA-seq analysis.

Compared with bulk-sample RNAseq, scRNA-seq data have high dropout rates (Hicks et al., 2018). Dropout rates are the opposite of gene counts, that is, the number of genes detected in a cell, a feature that is associated with cell differentiation status (Gulati et al., 2020). Variability in gene counts may cause imbalanced representation of non-expressed genes in gene signatures, resulting in scoring bias. Thus, we reason that in contexts where cells differ in differentiation status, failing to account for this variability may lead to misleading signature scores.

Cancer cells exhibit stem-cell like properties Malta et al., 2018; thus, cancer may represent such a context. To test this, we benchmark five signature-scoring methods in cancer single-cell datasets. The five methods included bulk-sample-based methods single sample gene set enrichment analysis (ssGSEA) and Gene Set Variation Analysis (GSVA) (implemented in the R GSVA package Hänzelmann et al., 2013) and three single-cell-based methods, AUCell (Aibar et al., 2017), Single-Cell Signature Explorer (SCSE) (Pont et al., 2019), and Jointly Assessing Signature Mean and Inferring Enrichment (JASMINE), a new method we developed. We show that cancer cells consistently express more genes than normal cells, and this imbalance significantly biases results from ssGSEA and GSVA but largely spares single-cell-based methods. Our results caution against the use of bulk-sample-based methods in scRNA-seq analyses.

## Results

### Cancer cells demonstrate higher gene counts than normal cells

**Figure 1.**
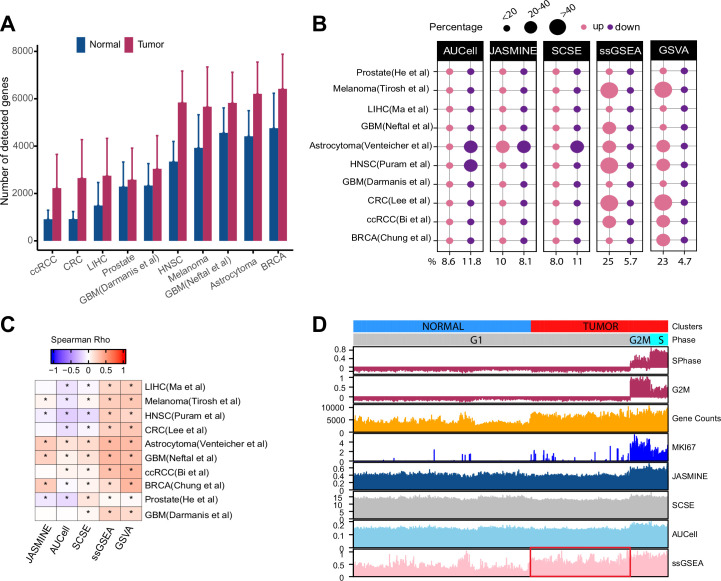
Gene count imbalances affect signature scoring. (**A**) The number of detected genes in tumor and normal cell populations in 10 single cell cancer RNAseq datasets. The height of each bar represents average, and whiskers represent standard deviation. In all cases, the difference is statistically significant (student t test, p < 2.2e-16). (**B**) Percentage of up and down regulated gene signatures in cancer cells relative to normal cells based on Cohen’s d. Dot size corresponds to the percentage of all signatures tested (*n* = 7503). (**C**) Spearman correlation coefficients of Cohen’s d with signature sizes across the datasets and methods. Asterisk (*) in each cell indicates p-value < 0.01. Color of the heatmap represents correlation coefficient. (**D**) Scores of a cell cycle gene set (GO:0007049) calculated using four methods along with MKI67 expression, gene counts, and cell cycle phases predicted by Seurat in Tumor and normal cell populations of HNSC dataset (GSE103322). The red box highlights non-cycling tumor cells that exhibit higher scores than non-cycling normal cells. Figure 1—source data 1.Source data for Figure 1.

Several recent studies showed that gene counts of single cells are associated with cell differentiation status and cell identity (Gulati et al., 2020; Qiu, 2020). Specifically, cells higher in the differentiation hierarchy express more genes. To examine if cancer and normal cells demonstrate similar imbalances, we collected 10 scRNA-seq datasets across different cancer types, technological platforms and sequencing coverage (Supplementary file 1). We also obtained cell type annotations from the original studies. We found that the average number of detected genes was significantly higher in tumor cells than in normal cells across all datasets (Figure 1A, p < 2.2e-16). This imbalance persisted when we separated normal cells into different cell populations (Figure 1—figure supplement 1). However, cancer-associated cells, including cancer-associated fibroblasts (CAF), tumor-associated macrophages (TAM), and tumor-related endothelial cells (TEC) had higher gene counts than other normal cell types, sometimes even comparable to malignant cells (Figure 1—figure supplement 1).

**Figure 1—figure supplement 1.**
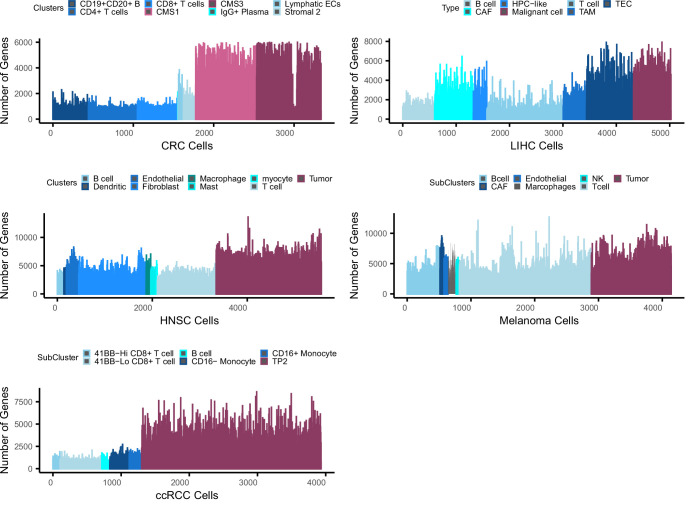
Bias in gene counts. Number of genes expressed in tumor and normal cell types across five single cell data sets including colorectal cancer (CRC), liver cancer (LIHC), head and neck cancer (HNSC), melanoma, and clear cell renal carcinoma (ccRCC). Maroon colors represent tumor cell populations in each data set while blue represents normal cell populations across the datasets. Note that in the LIHC dataset, CAF, TAM, and TEC are cancer-associated fibroblasts, tumor-associated macrophages, and tumor-related endothelial cells, respectively.

**Figure 1—figure supplement 2.**
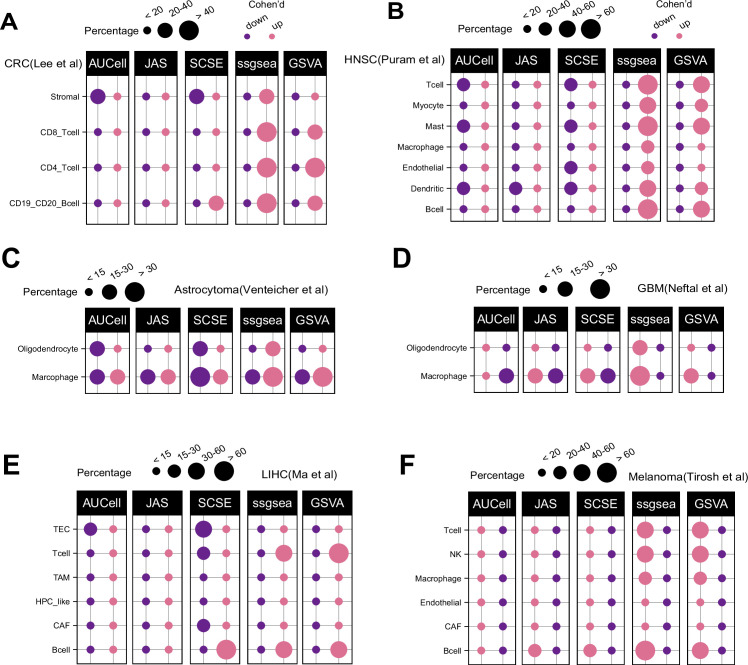
Patterns of up and down regulated signatures. Comparing tumor and normal cell populations across six additional datasets, including (**A**) colorectal, (**B**) head and neck cancer, (**C**) astrocytoma, (**D**) IDHwt GBM, (**E**) liver, and (**F**) melanoma. The size of each dot represents the percentage of up or down signatures over all signatures tested (*n* = 7503).

**Figure 1—figure supplement 3.**
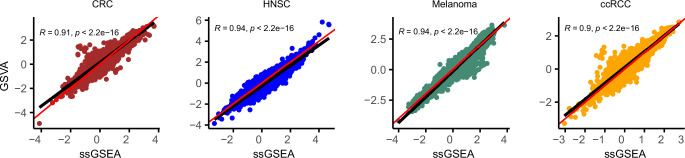
GSVA and ssGSEA comparison. Comparison of effect size (Cohen’s d) for ssGSEA (x-axis) and GSVA (y-axis) in four datasets: colorectal cancer (CRC) , head and neck cancer, melanoma, and clear cell renal carcinoma. Red line represents X = Y and black line is the regression line. Correlation is calculated using Spearman method.

### Bias of ssGSEA signature scores in cancer datasets

The reliability of signature-scoring methods is measured by their ability to identify the quantitative differences between two groups of samples at the gene-signature level. Here, we compare signature scores between tumor and normal cells. One issue is that scores from these methods differ in range and variance, thus making direct comparison challenging. To overcome this challenge, we used Cohen’s d (Cohen, 1988), a measurement that normalizes mean differences with standard deviation, thus generating unitless contrast that can be compared across methods. For simplicity, we defined Cohen’s d greater than one as upregulated, and less than –1 as down regulated. These criteria were used throughout this work.

We assembled 7503 gene sets from MSigDB that each has at least 20 genes (The term ‘gene set’ is used interchangeably with ‘gene signature’; Methods). On average, single-cell methods reported similar proportions of up- and down-regulated gene sets (9% vs 12% of all input gene sets) (Figure 1B). In contrast, ssGSEA and GSVA reported 25 and 23% of the input gene sets as upregulated but only 6 and 5% as down regulated. In six out of the 10 datasets, ssGSEA and GSVA estimated more than twice as many upregulated gene sets than down gene sets. This pattern remained when we divided normal cells into different populations (Figure 1—figure supplement 2). Notably, in cell types with high gene counts such as TEC, TAM, and CAF, we did not observe significantly more upregulated gene sets by ssGSEA and GSVA (Figure 1—figure supplement 2E,F). These results collectively suggest ssGSEA and GSVA are sensitive to variability of gene counts common to scRNA-seq data.

We also found that Cohen’s d from ssGSEA and GSVA showed consistent positive correlation with gene set sizes (Figure 1C). Single-cell methods did not demonstrate this pattern.

We observed that GSVA was slower than other methods. For instance, running the head and neck dataset with 200 threads on a 2.5 GHz Xeon processor took more than 3 days to complete. Because GSVA outputs were highly consistent with those of ssGSEA (Figure 1—figure supplement 3), and its running speed would not likely scale up to single cell datasets, we dropped GSVA from subsequent analyses.

We next use an example to illustrate how gene counts may affect signature scores and data interpretation. We scored the cell cycle gene signature from Gene Ontology (GO:0007049) in the head and neck cancer (HNSC) dataset (Puram et al., 2017). For comparison, we used Seurat (Butler et al., 2018) to identify cycling cells at G2M and S phases and also included the expression of KI67, a proliferation marker expressed by cycling cells. We observed all four methods reported a higher score in cycling cells (Figure 1D). However, ssGSEA scores were significantly higher in non-cycling cancer cells than in normal cells although neither are cycling (Student’s *t* test, p < 2.2e-16). In contrast, the single-cell-based methods did not show this difference.

### Sensitivity and specificity

We next generated gold standard up- and down-regulated gene sets at various sizes to benchmark detection sensitivity. To simulate real-world scenario, we added noises to each gene set so that higher noise levels attenuate more of their signals (Method).

**Figure 2.**
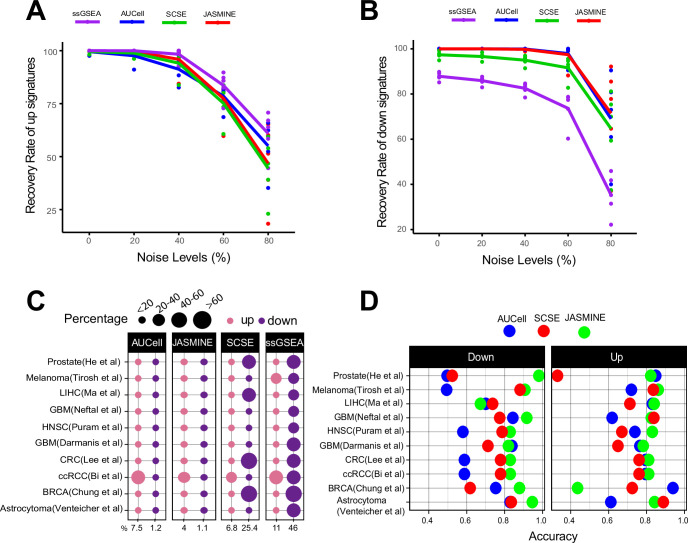
Sensitivity, specificity and accuracy. (**A**) Recovery rate for up gene signatures across five noise levels by the four methods. Each dot represents one dataset. At each noise level, average of all datasets is used to represent the performance of each method. (**B**) Similarly, for down signatures. (**C**) Percentages of false up and down signatures. The size of the dots corresponds to the percentages of all the signatures tested. Because the contrasting groups are generated by down sampling, no signatures are expected to be identified. The numbers below the heatmap are the average percentage. (**D**) Accuracy of the three methods, separated into up and down signatures. Accuracy is calculated as the agreement with consensus calls by at least two methods. Figure 2—source data 1.Source data for Figure 2.

We found gene set size had negligible impacts on detection rates (Figure 2—figure supplement 1). Noise levels, on the other hand, had a much bigger impact. For up gene sets, all methods were able to detect 50% of the gene sets on average even at the 80% noise level (Figure 2A). However, for down gene sets, ssGSEA performed worse than other methods (Figure 2B). Without noise, it only detected about 85% of the gene sets. At 80% noise level, it detected around 30% of the gene sets, compared to 70–80% by single-cell methods.

**Figure 2—figure supplement 1.**
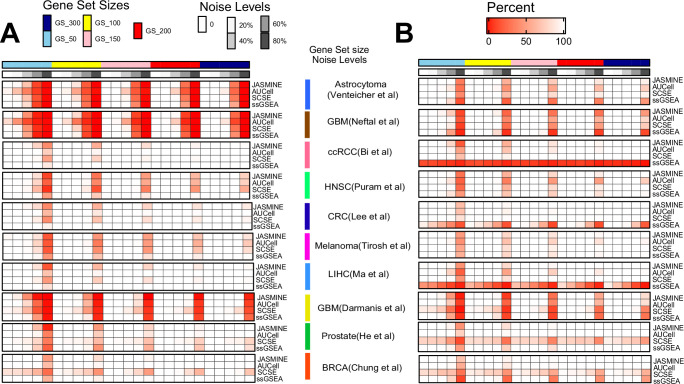
Benchmarking sensitivity using simulated gene signatures. We simulated four gene set sizes (50, 100, 150, 200, and 300), each with five levels of noise (0, 20, 40, 60, and 80%). For each size/noise combination, we randomly generated 1000 signatures. The results shown in this figure are percentage of the 1000 random signatures. (**A**) Detection sensitivity for up gene signatures. Deeper color indicates lower recovery rates (thus more misses). (**B**) Detection sensitivity for down signatures.

**Figure 2—figure supplement 2.**
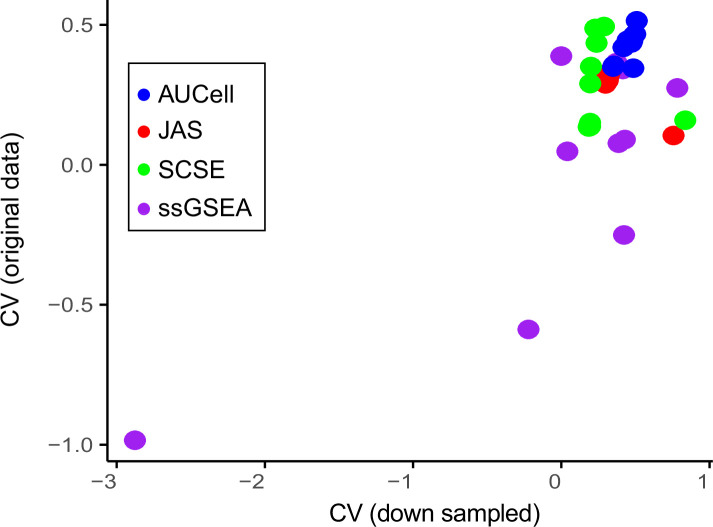
Coefficient of Variance. Average coefficient of variance between the original datasets and the 50% down-sampled datasets. Each dot represents one dataset.

**Figure 2—figure supplement 3.**
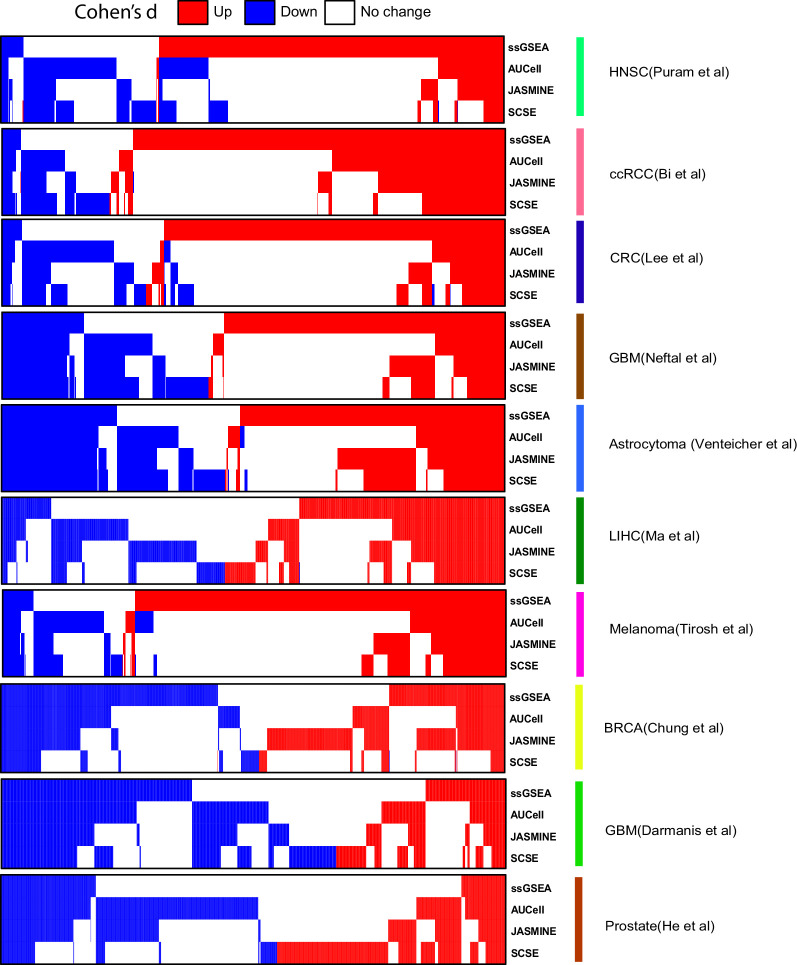
Comparison of calling results from the four methods across the seven datasets. In heatmap, each column represents one signature. Blue, down signature; red, up signature.

**Figure 2—figure supplement 4.**
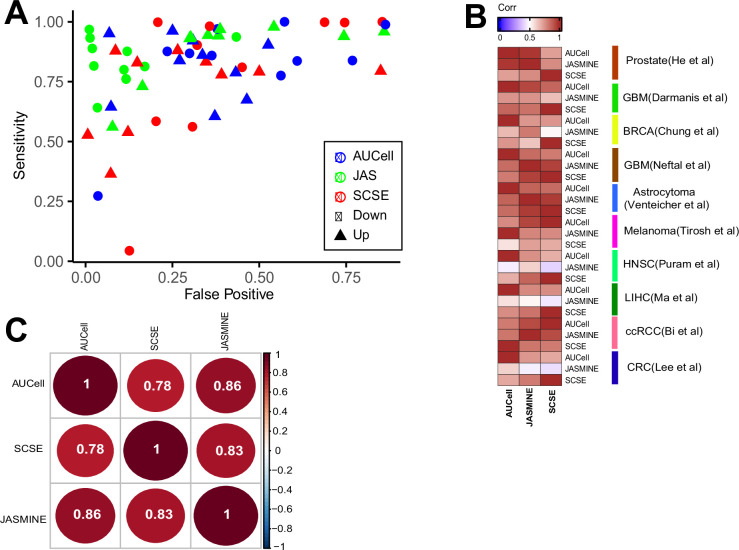
Consistency with consensus and pairwise comparison. (**A**) Sensitivity and false positive benchmarked against the consensus calls (signatures called by at least two methods). (**B**) Spearman correlation of Cohen’s d broken down to each dataset. (**C**) Consistency between three methods, numbers are Spearman correlation coefficients.

**Figure 2—figure supplement 5.**
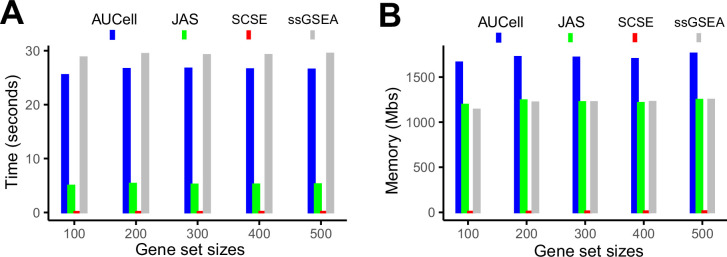
Evaluation of computing cost. (**A**) Average time consumption for completing 50 gene signatures using a 2.2 GHz, 32 GB memory CPU. (**B**) Memory cost for completing 50 gene signatures using a 2.2 GHz, 32 GB memory CPU.

To benchmark detection specificity, we generated null datasets by randomly choosing 100 cancer cells from each dataset. We down sampled each cell to 50% coverage, resulting in lower gene counts. These cells were then normalized to the same total coverage. Because the down-sampled cells were the same cells with the original, no up or down gene sets were expected between the two groups.

We found AUCell and JASMINE outperformed SCSE and ssGSEA in specificity (Figure 2C). On average, AUCell detected 7.5 and 1.2% of the input gene sets as up and down; JASMINE detected 4 and 1%. In four datasets, SCSE overestimated down regulated gene sets (average 25.4%). ssGSEA grossly overestimated both up- and down regulated gene sets in all datasets (average 11 and 46%, respectively).

Next, we estimated how changes in gene count affected score stability. For each gene signature, we calculated coefficient of variance (CV) (Figure 2—figure supplement 2). We observed that single-cell methods were robust to our down sampling, with little changes in CV. However, in three datasets, ssGSEA had a higher CV in down-sampled cells than in original cells.

### Comparing with consensus and computational efficiency

We next compared the three single-cell-based methods against consensus—gene sets that were identified as up- or down regulated by at least two methods. JASMINE aligned better with the consensus in most datasets (Figure 2D and Figure 2—figure supplement 3). AUCell overall had lower accuracies against the consensus, particularly for down regulated gene sets, likely because it is designed to score marker signatures (Figure 2D). When breaking down to sensitivity and specificity, AUCell showed higher false positive rates (Figure 2—figure supplement 4A). The three tools showed comparable sensitivity. Across the 10 datasets, SCSE and JASMINE showed better correlation (Figure 2—figure supplement 4B,C), thus explaining their relatively better alignment with the consensus.

Finally, we tested computing efficiency of the four methods. We randomly generated gene sets of various sizes and analyzed each gene set in a dataset consisting of 2000 cells. Figure 2—figure supplement 5 shows the time and memory use averaged over 50 iterations for one gene set under the same hardware configuration (2.20 GHz CPU, 32 GB memory). Overall, SCSE was the most efficient computationally. JASMINE was the second fastest algorithm but required a memory similar to that of ssGSEA. AUCell was slightly faster than ssGSEA but required the most memory. Their performances were robust across signature sizes.

### Dropouts affect ssGSEA scores

**Figure 3.**
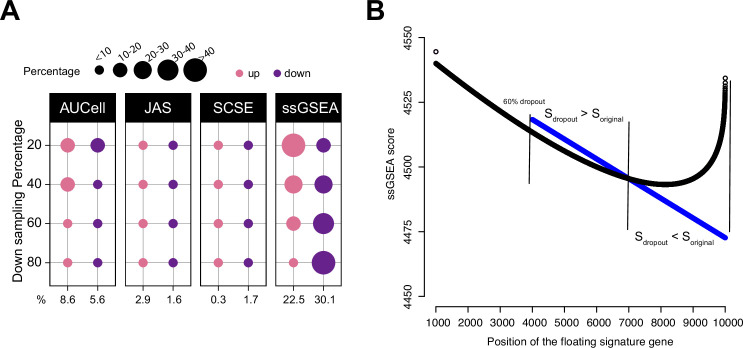
Impact of dropouts on ssGSEA signature scoring. (**A**) Percentages of up and down regulated gene signatures in original cells relative to down sampled cells for four levels of down sampling (20, 40, 60, and 80%) based on Cohen’s d. Dot size corresponds to the percentage of all signatures tested (*n* = 7503) in Head and Neck (Puram et al., 2017). (**B**) Effect of dropouts on ssGSEA scoring using a dummy expression matrix. The black line denotes the cell without any dropouts, and the blue line denotes the same cell with a 60% dropout rate. Note that for the gene signature, the first 99 genes are fixed. The x axis reflects the position of the last signature gene. When the gene is at rank <4000. The two cells give identical scores. However, after entering dropout zone, the scores start to deviate. Figure 3—source data 1.Source data for Figure 3.

To further show the impact of dropouts on ssGSEA scores, we re-ran the down sampling experiment by setting down sampling rates to 20, 40, 60, and 80%. A down sampling rate of 80% indicates that the total sequencing depth of the down sampled cell is at 80% of the original cell. A lower down sampling rate creates more dropouts. We observed that as the down sampling rate shifted from low to high, the pattern of deregulated gene sets by ssGSEA also shifted from more up gene sets towards more down gene sets (Figure 3A and Figure 3—figure supplement 1). This observation strongly associates dropouts with ssGSEA scores. In Figure 3—figure supplement 2, we provide an example using real data to show how ssGSEA scores change when limited to cells with comparable gene counts.

**Figure 3—figure supplement 1.**
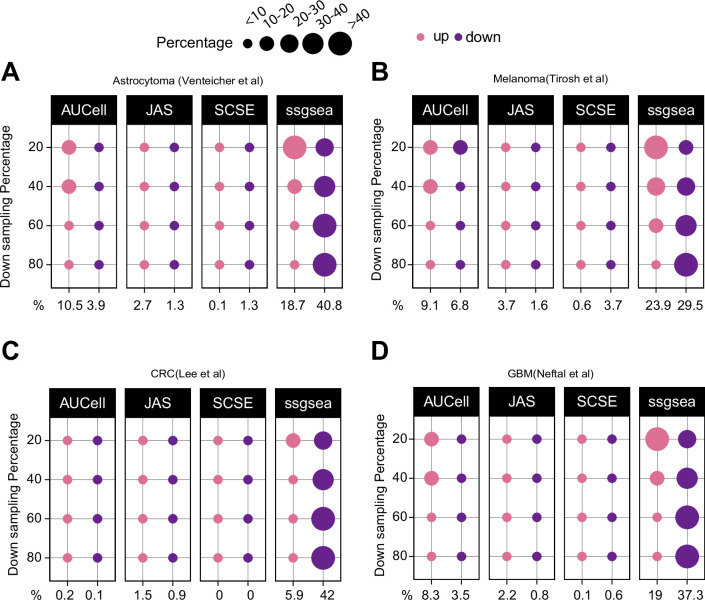
Down sampling levels affect signature scoring. Percentage of up and down regulated gene signatures in original cells relative to down sampled cells for four levels of down sampling (20, 40, 60, and 80%) based on Cohen’s d. Dot size corresponds to the percentage of all signatures tested (*n* = 7503) (**A**) in astrocytoma, (**B**) melanoma, (**C**) colorectal cancer, and (**D**) in glioblastoma data.

**Figure 3—figure supplement 2.**
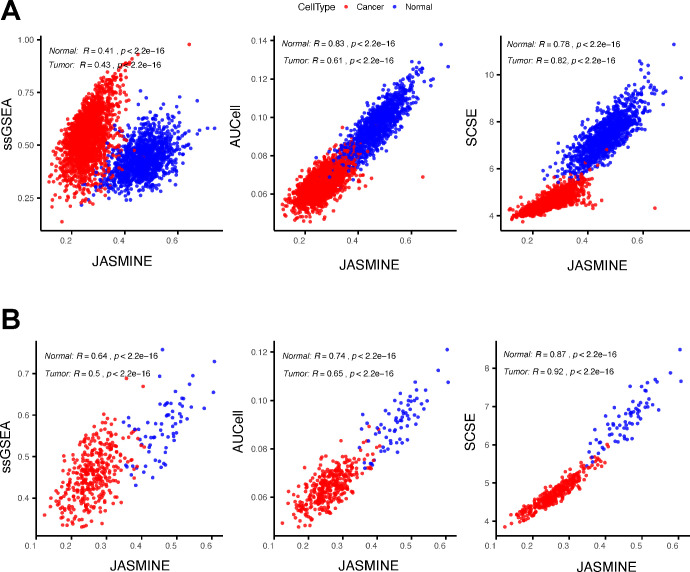
An example showing ssGSEA score changes. (**A**) Comparing scores of a gene signature (‘ZNF597_TARGET_GENES’) between JASMINE and other tools in all tumor and normal cells using the head and neck data. (**B**) The same comparison but limited to cells with the number of expressed genes between 4000 and 5000.

To understand how dropouts may affect ssGSEA scoring, we simulated a dummy expression profile of a cell with 10,000 genes. We then set different dropout rates to the cell ranging from no dropout to 80% dropout. Genes were ranked from high to low expression to mimic ssGSEA calculation and the gene rankings were identical at each dropout rate. We then assembled a gene signature of 100 genes. The first 99 genes were drawn from the top 1000 genes, and the last gene was added to the signature from the 1001st gene onward to the bottom of the list. This way, the signature consists of 99 highly expressed genes and a floating gene. As the floating gene moves toward the dropout zone, we can observe how the signature score changes, especially around the dropout zone. The codes to reproduce these data can be found at our Github repository (Methods). Figure 3B shows the result at the 60% dropout rate. Before entering the dropout zone, the signature score was identical between the no-dropout cell and the 60%-dropout cell. However, when the floating gene was between ranks 4000 and 7000, the signature was scored higher in the dropout cell; when moving further toward ranks 7000–10,000, the signature was scored lower in the dropout cell. Thus, the scores teetered around the tie rank of dropout genes, that is, 7000. This simple experiment demonstrates that though tedious, dropouts are sufficient to change ssGSEA scores in unintended ways.

## Discussion

In this study, we benchmarked five signature-scoring methods, including two that were developed for bulk sample RNAseq analysis (ssGSEA and GSVA), and three that were developed for scRNA-seq analysis (AUCell, SCSE, and JASMINE). Because ssGSEA and GSVA generate highly correlated scores and GSVA is slower, we focused on ssGSEA and the other three methods. Unlike methods that test for signature enrichment between sample groups, these signature-scoring methods quantify expression activity of a gene set in a sample independent of other samples. Outputs from these methods can be conveniently tested for association with complex sample characteristics. We show that single-cell-based methods are more robust at identifying *bona fide* up- and down regulated gene signatures from scRNAseq data. In contrast, bulk-sample-based ssGSEA and GSVA are more susceptible to dropouts. These disparities stem from their distinct mechanism to score signatures. AUCell uses Area Under the Curve (AUC) to test the enrichment of a gene set among top expressed genes in a cell. SCSE measures a signature using normalized total expression of the signature genes. AUCell and SCSE intuitively counter dropout effects by using only expressed genes. JASMINE considers the enrichment of signature genes in expressed genes to counter dropout effects, and meanwhile, evaluates the average expression level of the expressed signature genes. Bulk-sample-based methods were not designed to deal with excessive dropouts, which may mislead analysis especially if cell biology underpins different dropout rates. We further show that among single-cell methods, JASMINE and SCSE showed better concordance, and JASMINE outperforms SCSE in down sampling tests.

Dropouts were initially thought to result from low amounts of input RNA and transcriptional stochasticity. However, more studies showed that dropouts are associated with cell states and identities (Gulati et al., 2020; Qiu, 2020). Thus, in cellular contexts where cells may have systematic differences in dropout rates, any tool that fails to account for dropouts may generate misleading even erroneous data. In conclusion, our results caution against using bulk-sample-based signature-scoring methods to score single cells. Our study also suggests that it is important to consider cellular contexts when benchmarking methods for scRNA-seq data analysis, particularly when bulk-sample-based methods are involved.

## Materials and methods

### Single cell data sets and signature scoring

We used single cell data sets from 10 published studies (Bi et al., 2021; Chung et al., 2017; Darmanis et al., 2017; He et al., 2021; Lee et al., 2020; Ma et al., 2019; Neftel et al., 2019; Puram et al., 2017; Tirosh et al., 2016; Venteicher et al., 2017) for the evaluation of number of expressed genes in tumor versus normal cells to identify significant heterogenous patterns among the two phenotypes. Annotations of cell identity were also downloaded from each publication. We filtered all the data sets by removing non-expressed genes and then applied regularized negative binomial regression implemented in Seurat for normalization. We used C2 (*n* = 6226), C3 (*n* = 3556) and Hallmarks (*n* = 50) modules from MSigDB (Subramanian et al., 2005) v.7.2, to calculate the ratio of signature genes across all data sets and further signature scoring. We tested five tools for signature score calculations, including SCSE (Pont et al., 2019), AUCell (Aibar et al., 2017), ssGSEA, GSVA (Hänzelmann et al., 2013), and JASMINE. GSVA was included in tumor-normal comparisons but was dropped in gold standard tests and down sampling experiments due to slow running speed and highly correlated outputs with ssGSEA. We used GSVA and ssGSEA methods implemented in the GSVA Bioconductor (Hänzelmann et al., 2013) and AUCell method from AUCell Bioconductor packages (Aibar et al., 2017) with default parameters. We implemented SCSE in the R environment (v4.0) according to the equation reported in their paper (Pont et al., 2019). The output scores were used as is in tumor/normal cell comparisons and simulation analyses.

### **J**ointly Assessing Signature Mean and Inferring Enrichment

For each signature, JASMINE calculates the approximate mean using gene ranks among expressed genes and the enrichment of the signature in expressed genes. The two are then scaled to 0–1 and averaged to result in the final JASMINE score.

Assume *R_g_* represents the rank of an expressed signature gene *g* among genes with expression value >0 in a cell, then a mean rank vector *V_mean_* is calculated as follows:

$$V_{mean}=\sum_{i=1}^{m}R_{g,i}/(m\times N)$$

Where *m* represents the total number of expressed signature genes and *N* represents the total number of expressed genes in the cell. Because the mean is based on ranks, it is robust to scale normalization methods. It assesses the expression level of the signature only using genes detected in one cell, thus minimizing the effect of dropouts.

To assess signature enrichment in the expressed genes, we calculate either the Odds Ratio (OR) using four variables: a = signature genes expressed, b = signature genes not expressed, c = non-signature genes expressed, and d = non-signature genes not expressed. The signature enrichment using OR is calculated as follows:

OR=(a*d)/(b*c)

In practice, *c* is unlikely zero. For smaller signatures, b can be occasionally 0. In that case, we replace it with 1. In our testing, we observed that 99% of the gene sets did not encounter this issue in any of the cells we analyzed. Despite this rare incidence, we provide Likelihood Ratio (LR) as an alternative to OR in the JASMINE function. LR is calculated as follows:

$$LR=a*(c+d)/(c*\left(a+b\right))$$

*OR* and *LR* generate highly similar results according to our tests. *OR* (or *LR*) assesses the distribution of signature genes against the dropout background. Finally, *V_mean_* and *OR* are linearly scaled to [0–1] and are averaged to generate JASMINE scores.

### Effect size for gene sets scores

We first filtered the 9835 gene sets to 7503 by requiring a minimum size of 20 genes. We scored these gene sets (C2, C3, and hallmarks) among tumor and normal phenotypes using Cohen’s d (Cohen, 1988). We utilized an R package ‘effectsize’ (Ben-Shachar et al., 2020) to calculate this metric. We used Cohen’s d ≥ one as a threshold for positive or up cases and d ≤ –1 as negative or down cases with respect to tumor versus normal cells.

### Gene signature simulation

We first identified differentially expressed genes for each dataset using MAST (Finak et al., 2015), a method that explicitly accounts for dropout rates. We then randomly drew *N* genes from upregulated genes to generate an up gene set of size *N*, and similarly for down gene sets. Because in practice up and down regulated gene sets contain genes that have no expression change, or even changes at opposite directions, we added noises to the simulated gene sets. For instance, when setting noise level to 20%, an up gene set of size *N* would have 20% of its genes drawn from the remainders other than the upregulated genes. Following this procedure, we generated gene sets at the sizes of 50, 100, 150, 200, and 300 genes. For each size, we set the noise levels to 0, 20, 40, 60, and 80%. For each noise-size combination, we randomly generated 200 gene sets. In total, 5000 gene sets were generated per data set for each direction.

### Down sampling

We subset 100 tumor cells per data set for down sampling. The R package ‘scuttle’ (McCarthy et al., 2017) was used to down sample each cell to 50% total coverage. The down sampled data sets were then scaled back to ensure equal total coverage before comparison. Down sampling was also performed at various levels (20, 40, 60, and 80%) to examine its impact upon scoring of different methods especially ssGSEA.

### Dropouts impact on ssGSEA

We generated a toy example to demonstrate the impact of dropouts on ssGSEA scores. We used a signature comprised of n + 1 genes. We randomly selected 99 genes from top 1000 genes of the dummy expression matrix. These 99 genes are fixed. Then starting from 1001 onward, we added one gene to the signature each time, starting from 1001st gene index throughout the remaining list. This allowed us to investigate how signature score changes as the additional gene gradually moves from high expression to dropouts. We simulated the scenario with 0% (original scores), and 60% dropout rates. This simulation clearly reflects how the dropout rates significantly affect the scoring by ssGSEA.

### Data availability

Single cell data sets used in this study including their downloading sources were listed in Supplementary file 1. Gene sets were downloaded from MSigDB v.7.2 (http://www.gsea-msigdb.org/gsea/msigdb/index.jsp) for C2, C3 and Hallmark modules. JASMINE and ssGSEA testing codes are available on Github (https://github.com/NNoureen/JASMINE, copy archived at swh:1:rev:ba00996ad165ff471c6fada83e6cf76af50acdfa; Noureen, 2021b).

## Data Availability

The current manuscript is a computational study, so no data have been generated for this manuscript. Single cell data sets used in this study including their downloading sources were listed in Supplementary file 1. Gene sets were downloaded from MSigDB v.7.2. JASMINE source code is available on Github (https://github.com/NNoureen/JASMINE, copy archived at swh:1:rev:ba00996ad165ff471c6fada83e6cf76af50acdfa). Source Data contain the numerical data used to generate the figures. The following dataset was generated: NoureenN 2021Signature-scoring methods developed for bulk samples are not adequate for cancer single-cell RNA sequencing dataGitHubGitHub10.7554/eLife.71994PMC891677035212622 The following previously published datasets were used: TiroshI IzarB PrakadanSM 2016Single-cell expression of metastatic melanomaNCBI Gene Expression OmnibusGSE72056 PuramSV TiroshI ParikhAS PatelAP 2017Single-cell expression of head and neck cancerNCBI Gene Expression OmnibusGSE103322 BiK MxHe BakounyZ KanodiaA 2021Single-cell expression of advanced renal cell carcinomaBroad Single Cell portalSCP1288 LeeHO HongY EtliogluHE ChoYB 2020Single-cell expression of colorectal cancerNCBI Gene Expression OmnibusGSE144735 MaL HernandezMO ZhaoY MehtaM 2019Single-cell expression of liver cancerNCBI Gene Expression OmnibusGSE125449 NeftelC LaffyJ FilbinMG HaraT 2019Single-cell expression of IDH wildtype glioblastomaBroad Single Cell portalSCP393 VenteicherAS TiroshI HebertC YizhakK 2017Single-cell expression of IDH mutant astrocytomaBroad Single Cell portalSCP50 DarmanisS SloanSA CrooteD MignardiM 2017Single-cell expression of glioblastomaNCBI Gene Expression OmnibusGSE84465 ChungW EumHH LeeHO LeeKM 2017Single-cell expression of breast cancerNCBI Gene Expression OmnibusGSE75688 MxHe CuocoMS CrowdisJ Bosma-MoodyA ZhangZ BiK KanodiaA 2021Single-cell expression of prostate cancerBroad Single Cell portalSCP1244

## Additional files

Supplementary file 1. Single cell RNAseq datasets. The table lists all the ten datasets used in this study. Cell annotations were downloaded from the original publications.

Transparent reporting form
