# Supplementary material for: Signature-scoring methods developed for bulk samples are not adequate for cancer single-cell RNA sequencing data
Source: eLife. 2022 Feb 25;11:e71994. doi: 10.7554/eLife.71994 (PMC8916770; doi:10.7554/eLife.71994)
Supplement: Peer review history

# Editor report: Editor's evaluation

**DOI:** 10.7554/eLife.71994.sa0

**Authors:** C Daniela Robles-Espinoza

**Affiliations:** International Laboratory for Human Genome Research Mexico

In this revised manuscript, Noureen and collaborators benchmark five methods that are used in single-cell RNA sequencing data analyses, including single sample gene set enrichment analysis (ssGSEA), Gene Set Variation Analysis (GSVA), AUCell, Single Cell Signature Explorer (SCSE), and a newly developed method, Jointly Assessing Signature Mean and Inferring Enrichment (JASMINE). The authors test these in distinct cancer datasets and conclude that caution should be exercised when using bulk sample-based methods in single-cell data analyses, and cellular contexts should be taken into consideration. As a rapidly developing field in need of method benchmarking, we believe that this paper will be of great interest to the bioinformatics and single-cell data analysis communities.

# Decision letter

**DOI:** 10.7554/eLife.71994.sa1

**Affiliations:** International Laboratory for Human Genome Research Mexico; https://ror.org/03gds6c39 The University of Texas Health Science Center at Houston McGovern Medical School United States

Our editorial process produces two outputs: (i) public reviews designed to be posted alongside the preprint for the benefit of readers; (ii) feedback on the manuscript for the authors, including requests for revisions, shown below. We also include an acceptance summary that explains what the editors found interesting or important about the work.

**Decision letter after peer review:**

Thank you for submitting your article "Signature-scoring methods developed for bulk samples are not adequate for cancer single-cell RNA sequencing data" for consideration by *eLife*. Your article has been reviewed by 3 peer reviewers, and the evaluation has been overseen by a Reviewing Editor and Patricia Wittkopp as the Senior Editor. The following individual involved in review of your submission has agreed to reveal their identity: Leng Han (Reviewer #3).

The reviewers have discussed their reviews with one another, and the Reviewing Editor has drafted this to help you prepare a revised submission.

In this manuscript, Noureen and collaborators benchmark four methods that are used in single-cell RNA sequencing data analyses, including single sample gene set enrichment analysis (ssGSEA), AUCell, Single Cell Signature Explorer (SCSE), and a newly developed method, Jointly Assessing Signature Mean and Inferring Enrichment (JASMINE). The authors test these in distinct cancer datasets and conclude that caution should be exercised when using bulk sample-based methods in single-cell data analyses, and cellular contexts should be taken into consideration. Although interesting and informative, reviewers have raised a number of essential considerations that need to be addressed before publication.

Essential revisions:

Major Points

1. A reviewer comments, "The main analysis performed by the authors was identifying gene sets that significantly distinguish between cancer cells and the rest of the cells in the tumor. This a problematic comparison, since the cancer cells are epithelial cells (in most of the studies used) and the "normal" cells are stromal cells, mostly immune. Those are not comparable "normal" cells, and therefore it is expected that all immune-related pathways will be significant. The authors find much more down-regulated gene sets in ssGSEA compared to the other methods, but why are they wrong? If they are all immune related, I would actually conclude that ssGSEA is better than the other methods." They continue, "The comparison of cancer cells to normal microenvironment cells is meaningless. If looking at cancer, a relevant comparison would be cancer cells and normal adjacent cells. But cancer in general is not the best, as there would be way too many significant signatures, and its hard to understand what makes sense and what not". – Can the authors address this crucial observation please?

2. Can the methodology by which each method identifies signatures be explained? This would help identify why these methods perform differentially. Specifically, reviewers strongly suggest understanding what in ssGSEA makes it output different results. Gene dropouts could be the root cause, but this is something that needs proof. Empirically, reviewers suggest that this can be studied by comparing different scRNA-seq methods with different gene dropouts rates – compare 10X to SMART-seq. Do the authors have an idea of how this affects results?

3. The new method the authors propose (JASMINE) is not thoroughly explained and includes formulations which, in a reviewer's evaluation, lead to unintended mathematical behavior and hamper interpretation. Can the explanation on this method be expanded, please? Specifically, reviewers have raised the following points:

a. The four variables (a,b,c,d) considered when calculating the odds ratio make sense. However, the authors claim that "For smaller signatures b can be occasionally 0. In that case we replace it with 1." In a reviewer's words, "this is not a coherent and principled approach. I recommend the authors reconsider the mathematical formulation of this quantification to avoid the necessity of this unprincipled workaround."

b. A reviewer commented that "It is not clear why the average is the best way to combine the V_mean_ and OR to obtain the JASMINE score". Can this be specified, please?

[Editors’ note: further revisions were suggested prior to acceptance, as described below.]

Thank you for submitting your article "Signature-scoring methods developed for bulk samples are not adequate for cancer single-cell RNA sequencing data" for consideration by *eLife*. Your article has been reviewed by 3 peer reviewers, and the evaluation has been overseen by a Reviewing Editor and Naama Barkai as the Senior Editor. The following individual involved in review of your submission has agreed to reveal their identity: Leng Han (Reviewer #3).

The reviewers have discussed their reviews with one another, and the Reviewing Editor has drafted this to help you prepare a revised submission.

In this revised manuscript, Noureen and collaborators benchmark five methods that are used in single-cell RNA sequencing data analyses, including single sample gene set enrichment analysis (ssGSEA), Gene Set Variation Analysis (GSVA), AUCell, Single Cell Signature Explorer (SCSE), and a newly developed method, Jointly Assessing Signature Mean and Inferring Enrichment (JASMINE). The authors test these in distinct cancer datasets and conclude that caution should be exercised when using bulk sample-based methods in single-cell data analyses, and cellular contexts should be taken into consideration. The majority of the reviewers have found the answers to their initial points to be thorough and satisfactory, and so we would only ask the authors to address the points of one new reviewer, who replaced a previous reviewer for this round of revisions.

Essential revisions:

1. It is unclear how calibrated any of these methods are from the analysis presented in this manuscript. To help clarity, please address:

a) Can the authors please detail, in the Methods section, which thresholds are used on each program?

b) Is any test statistic is derived from the JASMINE method?

c) How is the final statistic is derived? That is, what is the range that the statistic is scaled by? In particular, in the eyes of a reviewer, the math for V_mean_ is unclear. They write, "As written, it is computed only over a fixed index g? The range on the sum is a bit confusing as well. Shouldn't it be over all the cells? If I were to guess, V_mean_ appears to be V_mean_ = \sum_{g=1}^{m}\sum_{c=1}^{total cells} R_{g,c}/(m*N). Is this correct?". Please clarify these points in the Methods section.

2. This manuscript infers that the main advantage of methods specific for single-cell data is to account for dropouts. However, in the view of a reviewer, the work presented here does not explicitly compare the differences between the bulk and single-cell methods, and they note that there might be factors that influence the results besides dropouts in real data. They argue that it is inconclusive to attribute poor performance of bulk methods to dropouts, especially when there is only one bulk method included in the analysis. For example, it is unclear how the authors ran ssGSEA, as there are some missing details in the Methods section. In particular, it is common practice to 'pre-filter' RNA-seq data before running any analysis on it. Did the authors do this for the real data analysis? Please add an introduction to each method, and add the details missing so readers can recapitulate the test conditions.

3. From Figure 1D, the authors reveal that the non-cycling tumor cells exhibit higher scores than non-cycling normal cells by ssGSEA. However, it is also noticeable that ssGSEA scores have a much higher variance than the other three methods, and results from SCSE and AUCell scores do not show a significant difference between cycling and non-cycling cells. Are there any explanations and/or intuition for such a result?

4. In the "Dropouts affect ssGSEA scores" section (Line 167), the "dummy expression matrix of 1000 genes and nine columns" is not clearly described. Is it for each cell? Is the ranking of genes the same for each dropout rate? Besides, Figure 3B is not fully explained: Could the authors please explain why the black line has a curvy feature towards the end of x axis while the blue line is linear? How about other dropout rates? There should be nine lines in the figure according to the matrix.

# Reply: Author response

**DOI:** 10.7554/eLife.71994.sa2

Essential revisions:

1. A reviewer comments, "The main analysis performed by the authors was identifying gene sets that significantly distinguish between cancer cells and the rest of the cells in the tumor. This a problematic comparison, since the cancer cells are epithelial cells (in most of the studies used) and the "normal" cells are stromal cells, mostly immune. Those are not comparable "normal" cells, and therefore it is expected that all immune-related pathways will be significant. The authors find much more down-regulated gene sets in ssGSEA compared to the other methods, but why are they wrong? If they are all immune related, I would actually conclude that ssGSEA is better than the other methods." They continue, "The comparison of cancer cells to normal microenvironment cells is meaningless. If looking at cancer, a relevant comparison would be cancer cells and normal adjacent cells. But cancer in general is not the best, as there would be way too many significant signatures, and its hard to understand what makes sense and what not". – Can the authors address this crucial observation please?

We agree with the reviewer that lineage is an important factor when comparing tumor and normal cells. Lineage specific signatures are expected to differ, but expectations are not reliable for benchmarking purposes because tumor cells may influence the expression of immune and stromal cells, and cancers of different lineages do not necessarily show the same differences with normal cells. A more objective approach is to compare outputs of each method with ground truth. This is why we designed two objective experiments to benchmark the reliability of the tested methods. In the first experiment, we generated gold standard up/down signatures and tested how well each method rediscovers them (sensitivity test, Figure 2a-b in initial manuscript). Note that generating these gold standard signatures does not rely on expectations, but rather on their constituent genes that are sampled from the pool of differentially expressed genes. In the second experiment, we generated pseudo-cells by down sampling. These cells are identical to the original cells in the distribution of expressed genes; thus, no signature should be identified. This experiment was to test how well each method eludes false positives (specificity test, Figure 2c in the initial manuscript). These experiments were designed precisely because of the challenges to establish functional gold standard, as the reviewer pointed it out.

We gently correct the reviewer that we found more up-regulated, not down-regulated gene sets in ssGSEA. We also respectfully remind the reviewer that in our manuscript, we never concluded that ssGSEA scores were wrong in the tumor vs normal comparisons. Instead, we concluded that “… ssGSEA is sensitive to variability of gene counts common to scRNAseq data” (lines 94-95 on page 4, first submitted version). We respectfully argue that the observation of many more deregulated signatures called by ssGSEA preludes the more systematic benchmark analyses we laid out later in the manuscript, because it substantiates the hypothesis that signature scores can be biased if a method does not factor in dropouts in single cell data.

To give the reviewer and readers an intuitive example of how ssGSEA scores may mislead single cell interpretation, we calculated the score of the cell cycle signature (Gene Ontology, GO:0007049). As the reviewer is aware, cycling cells often need to be regressed out before clustering in single cell analysis. In this example, we used the head and neck data (Puram et al., 2017), and normal/tumor cell annotations were downloaded from the publication. We divided cells into cycling (S phase, G2M phase) and non-cycling cells (G1) using Seurat. Very few normal cells were determined as cycling, and thus, were removed from the figure. We additionally added the expression of KI67 to further confirm cell cycle status. KI67 is an established proliferation marker and is expressed by cycling cells but not quiescent cells. As shown in the figure, KI67 expression agrees well with Seurat scores. Notably, in tumor cells highlighted in the red box, ssGSEA scores were significantly higher than those in normal cells, suggesting the tumor cells may be cycling. However, neither these tumor cells nor the normal cells are cycling according to other evidence. Thus, at the minimum, ssGSEA scores complicate the interpretation of cell cycle status in this example. None of the single-cell methods showed the same bias.

This example was included in our preprint but was omitted in our initial submission. In the revision, we have added it back to support our conclusion. We again apologize for the omission, and hope the reviewer finds that this example makes biological sense.

2. Can the methodology by which each method identifies signatures be explained? This would help identify why these methods perform differentially. Specifically, reviewers strongly suggest understanding what in ssGSEA makes it output different results. Gene dropouts could be the root cause, but this is something that needs proof. Empirically, reviewers suggest that this can be studied by comparing different scRNA-seq methods with different gene dropouts rates – compare 10X to SMART-seq. Do the authors have an idea of how this affects results?

We thank the reviewers for suggesting new analyses to better understand the impact of dropouts on signature scores. Comparing SMART-seq with 10x does reproduce the differences in dropout rates. Among the 10 datasets we used, five were from SMART-seq, four from 10X and one from C1 fludigm. However, one cell cannot be sequenced twice with two scRNAseq platforms. Therefore, it is unfeasible to compare data of the same cells generated by the two platforms. Alternatively, we can compare the same cell population sequenced by the two. In a recent study (PMID: 33662621), Wang et al., sequenced the same samples of CD45^-^ cells with both SMART-seq and 10x platforms. They showed that the RNA composition detected by the two platforms differ. Specifically, the 10x platform detected more ribosomal genes and lncRNAs, whereas SMART-seq detected more mitochondrial genes. The differences in RNA composition are critically important for RNA sequencing data analysis (see Robinson et al., Genome Bio, 2010. PMID: 20196867; and Zhao et al., RNA, 2020. PMID: 32284352), and thus, gene expression values are not directly comparable between the two platforms. For the same reason, gene signatures scores are not directly comparable either. Another confounding factor is cell-to-cell variation such as caused by transcriptional bursting.

Down sampling is an established method in single cell RNAseq analysis to recapitulate the effects of differences in sequencing depth. The method increases technical dropout rates and creates a realistic expression profile at a prespecified depth (Luecken M and Theis F, Mol Syst Biol, 2019. PMID: 31217225). Using this method, we can generate the expression profile of the same cell at a lower depth (conversely, higher dropout) without confounding effects from other factors. As we show in Figure 2C, at the 50% down sampling rate, ssGSEA identifies many deregulated signatures between the original cells and down sampled cells across all ten datasets. These concerning results indicate dropouts likely affect ssGSEA much more than other methods.

To further prove this, we set down sampling levels to 20%, 40%, 60% and 80%. Here, 80% down sampling means after the procedure, the cell is at 80% sequencing depth of the original cell. As down sampling level decreases, more dropouts are created as the procedure primarily affects genes with low counts. New Figure 3A shows how the number of up and down signatures identified by ssGSEA shifts along with down sampling levels. Because the dropout rate is the only parameter we manipulated in this analysis, the results clearly indicate that it impacts ssGSEA scores. In contrast, single-cell methods do not show the same correlation. We replicated the results in other datasets (see Figure 3—figure supplement 1 in the revised manuscript).

An empirical approach to this question is to take advantage of cell-to-cell variation and examine how scoring patterns change as cells with similar dropout rates are compared. In new Figure 3-figure supplement 2, the top panel shows score correlation of a signature between JASMINE and the other three methods using all cells from the head and neck data. Normal cells are scored higher by single cell methods but not ssGSEA, despite that JASMINE and ssGSEA scores are nicely correlated in both normal and tumor cells. In the bottom panel, we limited tumor and normal cells to those with 4,000-5,000 expressed genes. With this filtering, tumor cells still have higher gene counts than normal cells on average (tumor, 4585; normal, 4407), but the difference is much smaller than using the entire dataset. Nevertheless, using these cells led to a much more consistent pattern between ssGSEA and single-cell methods, further supporting our conclusion.

We agree with the reviewers that more insights are needed, particularly related to ssGSEA. The method scores a signature by summing up differences in the empirical cumulative density of genes inside and outside the signature. To do so, ssGSEA orders and ranks genes from high to low expression and iterate through every gene. For bulk expression data, the ranks are continuous; thus, signature scores gradually change depending on the distribution of the signature genes on the rank list. Single cell data often encompass thousands of dropouts. Because dropout genes have equal expression values, i.e., zero, their ranks are tied, thus disrupting the presumption of continuous data distribution. This predicates that if a signature has no gene in the dropout zone, the signature score is not affected by dropout rates. Otherwise, the score will be affected. We think this is an important issue, because using 10x, it is common to detect only 2,000-4,000 genes; even smart-seq usually only detect ~6,000 genes. It is thus highly likely that genes from gene signatures fall into the dropout zone. Single cell methods dodge this issue for various reasons. AUCell and SCSE do not use dropout genes in scoring, whereas JASMINE explicitly considers dropout rate in the calculation.

We next designed a computational approach to test this idea. We first generated a dummy expression vector with 10,000 genes. The genes were ranked from high to low based on expression, the same as ssGSEA. This expression vector mimics bulk expression data. Then we set the bottom of the vector to zeros to create dropouts at various rates. Next, we generated a highly expressed gene signature of 100 genes. This 100-gene signature consisted of 99 genes that were randomly sampled from the top 1000 genes, and the last gene (the floating signature gene) was iterated from the 1001^st^ gene onwards to the bottom of the gene list. This allowed us to observe how the signature score changes as the floating signature gene moved towards the dropout zone. We have provided the source code of this process in our Github repository for the reviewers and other researchers to reproduce our results and test different scenarios using the dummy expression data (https://github.com/NNoureen/JASMINE).

In new Figure 3B, the black line shows the signature score at no dropout. The blue line shows the signature score when the floating gene enters the dropout zone at 60% dropout rate. At this rate, the dropout zone is between the gene rank 4,000 and 10,000. Before the floating signature gene enters the dropout zone (at gene rank 4,000), the signature score is identical between the original cell and down sampled cell. However, between gene rank 4,000 and 7,000, the signature is scored higher in the dropout cell than the control cell, and the pattern is reversed after gene rank 7,000. Note that gene rank 7,000 is the tie rank for all dropout genes. Clearly, the scores deviate from the intended behavior in the dropouts.

In the revision, we have added new Figure 3A, new Figure 3—figure supplement 2 and new Figure 3B to the manuscript. We have also discussed the reasons why these methods perform differently.

3. The new method the authors propose (JASMINE) is not thoroughly explained and includes formulations which, in a reviewer's evaluation, lead to unintended mathematical behavior and hamper interpretation. Can the explanation on this method be expanded, please? Specifically, reviewers have raised the following points:

a. The four variables (a,b,c,d) considered when calculating the odds ratio make sense. However, the authors claim that "For smaller signatures b can be occasionally 0. In that case we replace it with 1." In a reviewer's words, "this is not a coherent and principled approach. I recommend the authors reconsider the mathematical formulation of this quantification to avoid the necessity of this unprincipled workaround."

b. A reviewer commented that "It is not clear why the average is the best way to combine the V_mean_ and OR to obtain the JASMINE score". Can this be specified, please?

a. When calculating odds ratio, the denominator is b*c, where *b* is the number of signature genes that are not detected, and *c* is the number of nonsignature genes that are detected (see Author response table 1 for reviewers’ convenience).

**Author response table 1.** A breakdown of signature genes in a single cell expression profile. The four numbers are used in signature enrichment calculation by JASMINE.

	Expressed	Not-expressed
Signature gene	*a*	*b*
Non-signature gene	c	d

b. In practice, *c* is unlikely zero. *b* would be zero if all genes in the signature are expressed. In this scenario, the signature is highly enriched in the expressed genes, and thus, should be given a high OR score. Imagine for a signature consisting of 50 genes, if all 50 genes are expressed in a cell, the signature is apparently highly enriched with expressed genes. Even if we assume one of the signature genes is not expressed, the enrichment pattern will be largely the same. We think adding a small number is a mathematical necessity without significantly changing the biological interpretation. Our approach is not so much different from other methods such as Haldane correction which would add a small number, such as 0.5, to each variable in the OR calculation.

We agree with the reviewer that this workaround may deviate scores from the formula for some signatures. To clarify how many signatures may be affected, the following Author response table 2 summarizes the number of signatures that have b=0 in none of the cells, 1 cell, 2 cells, or ≥3 cells. The number in the parenthesis indicates percentage. The total number of cells analyzed in each dataset is also given in the column name. On average, 99% of the signatures we analyzed did not encounter this issue in any of the cells in the ten datasets. Given that each dataset has hundreds to thousands of cells, this issue is extremely rare.

**Author response table 2.** Counts and frequencies of signatures with b=0 in 0, 1, 2 and ≥3 cells across datasets. The number of cells analyzed in each dataset is given in the parenthesis at the column name.

cell # (b=0)	Astrocytoma(n=6176)	BRCA(n=515)	IDHwtGBM(n=5742)	HNSC(n=5560)	GBM(n=3589)
0	7425 (98.96)	7438 (99.13)	7395 (98.56)	7194 (95.88)	7326 (99.84)
1	33 (0.44)	25 (0.33)	33 (0.44)	161 (2.15)	7 (0.1)
2	18 (0.24)	10 (0.13)	17 (0.23)	46 (0.61)	2 (0.03)
≥3	27 (0.36)	30 (0.4)	58 (0.6)	102 (1.36)	3 (0.04)
**cell # (b=0)**	**LIHC** **(n=5023)**	**CRC** **(n=3336)**	**ccRCC** **(n=3933)**	**Prostate** **(n=2170)**	**Melanoma** **(n=4645)**
0	7482 (99.72)	7496 (99.91)	7489 (99.81)	7493 (99.87)	7253 (96.67)
1	8 (0.11)	2 (0.03)	7 (0.09)	7 (0.09)	79 (1.05)
2	7 (0.09)	3 (0.04)	1 (0.01)	2 (0.03)	43 (0.57)
≥3	6 (0.08)	2 (0.03)	6 (0.08)	1 (0.01)	128 (1.71)

Inspired by the reviewer’s comments, we have provided likelihood ratio as an additional approach to odds ratio in the revision. The first likelihood calculates the risk of a signature gene being detected as *a*/(*a*+*b*), and the second likelihood calculates the risk of non-signature genes being detected as *c*/(*c*+*d*). The second likelihood essentially provides a background, and approximately equals the ratio between the numbers of expressed genes and non-expressed genes. The likelihood ratio is calculated as the ratio of the two likelihoods, i.e., LR=a*(*c*+*d*)/(c*(*a+b*)). This will effectively avoid the 0 denominator issue. In our testing, the likelihood ratio and odds ratio gave highly consistent results: effect size correlation was higher than 0.95 in four of the five datasets tested. The new function has been implemented in the JASMINE code.

In the revision, we have added more details to the method description, and clarified the extent to which b=0 may affect signature scores. The JASMINE code has also been updated on Github. We thank the reviewer for prompting us to improve the JASMINE function.

b. We cannot say the average is the best way to combine the two. As insight evolves, there might be better and more sensible approaches to signature scoring. During our analysis, we tested multiple ways, including arithmetic mean, geometric mean, and some other non-linear methods. After the tests, we felt equal contribution from the two score components makes best sense, largely because we do not have a good reasoning to overweigh one option over another.

[Editors’ note: further revisions were suggested prior to acceptance, as described below.]

Essential revisions:

1. It is unclear how calibrated any of these methods are from the analysis presented in this manuscript. To help clarity, please address:

a) Can the authors please detail, in the Methods section, which thresholds are used on each program?

When we calculated scores with each method, no thresholds were used. However, when we compared signature scores between tumor and normal cells, we used Cohen’s d > 1 as criterion for up regulation and < -1 for down regulation. These thresholds have been clarified in the main text and Methods.

b) Is any test statistic is derived from the JASMINE method?

JASMINE does not derive a test statistic, similar to other methods benchmarked in the study. These methods calculate a score for an input signature in a cell/sample. These scores can be compared across cells/samples for differences in signature expression. Compared with methods such as regular GSEA, these single sample methods do not rely on class labels, and thus, offer better flexibility for downstream analyses. In our revised manuscript, we have provided further context in Discussion.

c) How is the final statistic is derived? That is, what is the range that the statistic is scaled by? In particular, in the eyes of a reviewer, the math for V_mean_ is unclear. They write, "As written, it is computed only over a fixed index g? The range on the sum is a bit confusing as well. Shouldn't it be over all the cells? If I were to guess, V_mean_ appears to be V_mean_ = \sum_{g=1}^{m}\sum_{c=1}^{total cells} R_{g,c}/(m*N). Is this correct?". Please clarify these points in the Methods section.

We thank the reviewer for meticulously checking the formula, and yes, it is indeed a bit confusing. To make it clearer, we have revised it as follows:

Assume *R_g_* represents the rank of an expressed signature gene *g* among genes with expression value > 0 in a cell, then a mean rank vector *V_mean_* is calculated as follows:

$$Vmean=\sum_{g=1}^{m}\;R_{g,c}/(m*N)$$

The final score is defined as the arthemetic mean of the 2 signature components: signature mean defined by Vmean, and signature enrichment defined by odds ratio or likelihood ratio. Each component has a range of 0-1.

2. This manuscript infers that the main advantage of methods specific for single-cell data is to account for dropouts. However, in the view of a reviewer, the work presented here does not explicitly compare the differences between the bulk and single-cell methods, and they note that there might be factors that influence the results besides dropouts in real data. They argue that it is inconclusive to attribute poor performance of bulk methods to dropouts, especially when there is only one bulk method included in the analysis. For example, it is unclear how the authors ran ssGSEA, as there are some missing details in the Methods section. In particular, it is common practice to 'pre-filter' RNA-seq data before running any analysis on it. Did the authors do this for the real data analysis? Please add an introduction to each method, and add the details missing so readers can recapitulate the test conditions.

We appreciate the reviewer’s concern. We did pre-filter the single-cell RNAseq datasets by removing genes that were not detected in any cells. As for the running of these methods is concerned, we have run ssGSEA and GSVA using the R biocondutor package GSVA. We used the default parameters to run these methods. Similarly we ran AUCell using their package with default parameters. SCSE did not provide R codes, only a web application. For fair comparison, we implemented it in R according to the formula reported in their publication. These details have been added to the methods section. We have also included some discussions on each method in the revised manuscript.

3. From Figure 1D, the authors reveal that the non-cycling tumor cells exhibit higher scores than non-cycling normal cells by ssGSEA. However, it is also noticeable that ssGSEA scores have a much higher variance than the other three methods, and results from SCSE and AUCell scores do not show a significant difference between cycling and non-cycling cells. Are there any explanations and/or intuition for such a result?

We thank the reviewer for raising this great question, which prompted us to include gene counts in the figure. Visually, it is clear that ssGSEA scores are associated with gene counts, which we think further strengthens our conclusion. We have replaced the original Figure 1D with the new one in our revised manuscript.

4. In the "Dropouts affect ssGSEA scores" section (Line 167), the "dummy expression matrix of 1000 genes and nine columns" is not clearly described. Is it for each cell? Is the ranking of genes the same for each dropout rate? Besides, Figure 3B is not fully explained: Could the authors please explain why the black line has a curvy feature towards the end of x axis while the blue line is linear? How about other dropout rates? There should be nine lines in the figure according to the matrix.

The dummy expression matrix of 10,000 genes and nine columns reflect a scenario where the first column representing a cell that has no dropouts; from the 2^nd^ column to 9^th^ column is the same cell but with different drop out rates ranging from 10 % to 80 % with the step of 10%. The ranking of genes is identical in each column. We agree with the reviewer that stating 9 columns in the text may lead to confusion because we did not show 9 lines in the figure. The reason we only showed 60% dropout (blue line) is that we think having one line is sufficient to show the deviation in the dropout zone, whereas having multiple lines would make the figure harder to read, and importantly, unnecessary given that they are essentially repeating the same message. The codes for this similatuion were published in our Github (https://github.com/NNoureen/JASMINE). We respectfully encourage the reviewer to check them.

As for the curly tail of the ssGSEA scores in the cell without dropouts, that is how ssGSEA penalizes the genes at the bottom of the gene list as shown in our floating gene simulation. Thus, it appears ssGSEA penalizes the gene less for the signature score if the gene appears at the bottom of the list. In our experiment, we only changed the position of the last signature gene while keeping all other settings the same. When the ranking of the bottom genes are identical due to dropouts, the penalization became linear, showing the score as a straight declining line. We think these are compelling data to show how dropouts can affect ssGSEA scoring.

In the revision, we have edited the text to mitigate confusion. We thank the reviewer for pointing out this.
